# Empathy, Introversion and the OSCE. Reflections of a 2nd Year Medical Student

**DOI:** 10.15694/mep.2018.0000254.1

**Published:** 2018-11-13

**Authors:** Joshua Lui, Alexia Papageorgiou

**Affiliations:** 1 St George’s; 2 Univeristy of Nicosia Medical School

**Keywords:** clinical competence, objectively structured clinical examination (OSCE), personality, introversion, extraversion, remediation, medical student, undergraduate medical education

## Abstract

This article was migrated. The article was marked as recommended.

Clinical communication skills training is a key element of modern medical education as it has been shown to have multiple benefits in delivering healthcare. Various factors affect clinical communication and we believe that personality is a major one. As an introverted second year medical student who has recently failed his Objectively Structured Clinical Examination (OSCE) despite passing the written knowledge examination with flying colours, I reflect on my OSCE experience and try to understand the reasons that caused my failure. Methods for improving communication skills such as role plays, systematic desensitisation and remediation are suggested. Although there is one study that has shown that introverted students score lower in OSCEs compared to extroverted students, more research is needed in this area in order to draw meaningful conclusions. Therefore, a research study is suggested to look into the correlation between personality traits and OSCE results. If a correlation is indeed found, it may suggest that medical schools should provide additional support in communication skills for introverted students.

## Introduction

Communication between doctors and patients is one of the most important elements in delivering health care. Multiple studies have been conducted to show how good communication can enhance not only the patient-doctor’s relationship but also the clinical outcome of illnesses. ( Silverman, Kurtz and Draper, 2013) Hence, communication skills teaching is being emphasised and incorporated into the curriculum and assessment in many undergraduate medical schools nowadays. One of the key elements in communication skills is empathy- “the ability to understand and share the feelings of the patients.” It is a skill that’s particularly focused on in undergraduate medical training. A number of studies have been conducted to show the different benefits of showing empathy to patients in healthcare. These include improved clinical outcomes ( Pollak *et al*., 2011) and improved patient satisfaction. ( Mauksch *et al.,*2008)

However, apart from the clinical communication skills training given by the medical school, what other factors influence a medical student’s ability to show empathy? Is personality one of the factors that affect the way a medical student or doctor communicate with his/ her patients? As an introverted second year medical student, I recently failed my end of year OSCE and I’m forced to repeat the whole academic year, despite passing with flying colours in the written knowledge test. After a careful analysis of the OSCE feedback I received from multiple stations, it is believed that my low grade was mainly due to my failure to express empathy and building rapport with the simulated patients, which forms a significant proportion of my school’s assessment. In light of this, I intend to use the first part of this article to write a self reflection of my own personal experience for taking the OSCE as an introvert, and to evaluate my approach to this examination. Secondly, I will also be exploring the literature to search for related studies regarding personality of medical students and their OSCE results.

## Personal Reflection

Many medical schools now use the Objective Structured Clinical Examination (OSCE) as part of the assessments for their medical students. OSCEs use multiple stations of real life scenarios and simulated or real patients to assess a variety of skills in medical students, including clinical competence, problem solving and communication skills. OSCEs can be viewed as a “driving test” for medical students, where they can only progress if they are deemed “safe” and “competent” for handling these simulated real life scenarios. The second year OSCE at my school comprised of twelve different ten-minute stations, which involved both clinical skill and communication skill stations. Clinical skill stations examine students’ fluency and technique on performing clinical examinations, whilst communication skill stations involve stations on history taking, information giving and shared decision making, confidentiality, etc.

One of the communication skills our school really emphasised is empathy, which is a big challenge to me. The school expects students to pick up verbal and non-verbal cues from patients where they signify they are suffering, then to reply in a manner to show our empathy. There are a number of reasons why I found this particularly challenging. Firstly, as an introvert, I mostly enjoy spending time alone rather than interacting with others. Therefore, I always find it hard to appreciate the feelings and emotions of other people, especially when they are people that I am not acquainted with. Secondly, under the extremely intense examination condition my mind was preoccupied with asking the specific medical questions. I found that to genuinely feel for the simulated patients was an additional task that I could not focus on at the same time. Finally, at the back of my mind I have always known that the simulated patients are in fact actors or actresses. Hence, my subconscious mind refused to genuinely feel for them, like I would do for the real patients in hospitals.

Due to all these reasons, I decided that I would not spend extra effort to genuinely reply in an empathetic way for each different scenario. Instead, for my OSCE I decided to adopt the “more practical” approach where I would just use a “standard empathy phrase” all the way along to hopefully suit every single scenario. This approach appealed to me because I didn’t have to be in any contact with the patient’s and my feelings, which I find a challenge as an introvert. It allowed me to fully concentrate on biomedical aspects of the patients. Plus, I wouldn’t have to spend extra effort in generating different empathetic phrases every single time to suit each scenario. Unfortunately, I would only find out later that this was a devastating approach.

A standard phrase that I picked up from our communication skills teaching for showing empathy was, “Oh, that must be very difficult for you.” During one of the stations, I recognised a cue from the simulated patient and therefore I immediately and automatically replied using exactly the same phrase. However, it came as an utter shock to me when I later found out in my feedback, “candidate attempted to show empathy, but did not sound genuine enough.” Indeed, I did not feel any empathy for the simulated patient at that point. The phrase was simply “regurgitated” from my mouth only in attempt to fulfil what I thought was the school’s requirement. I honestly thought that I could score my points by saying that phrase, regardless of how genuine I was. Then I could move on to my next set of medical questions. I was totally wrong. I realised that showing empathy or saying the empathic phrase is one thing, but more importantly, the school was looking at how the students were able to build rapport with the patients. Regardless of what words I used, sincerity of the expression is in fact the key in building rapport.

I could have easily put on a show by deeply saddening my facial expressions. But I disliked the idea because I didn’t want to be someone that was not me. I believed that I am enrolled to a medical school not an acting school. By being too genuinely indifferent, I failed to show empathy and build rapport with the simulated patients and hence I failed my OSCE. Clearly, being indifferent to patients’ suffering in an OSCE communication skills station will not make me score. On the other hand, pretending to show empathy is against my personal philosophy either. Therefore, it seems that the only option that I can adopt is to be my true self, whilst at the same time to also genuinely feel for the patients’ suffering. So what can I do to achieve this?

As human beings, we hear numerous stories from different people we encounter everyday. Each time we react to each of these stories in a unique way but also to a different extent. When we connect with these stories, they have the power to directly influence our own emotions, which would then be reflected on our facial expressions or reactions. Conversely, there are other times when we cannot connect with the stories. We just don’t care. A person’s expression of empathy can be influenced by various factors, such as stress, his past experience, confidence or personality. However, I don’t believe that there exists a type of totally heartless and indifferent personality who never cares about anything happening around him. Instead, studies have identified that empathy is one of our innate attributes. ( Tavakol, Dennick and Tavakol, 2012) As human beings we all have the tendency to care about or feel for people around us. The problem is that our empathy is often masked by the daily stress or burn out that we deal with every day and we become unable to care for others. ( Park *et al*., 2015) All we have to do is to “activate” or “awaken” our own genuine empathy that is already installed in our genetics and to express it in our daily interactions. Some people are better at showing empathy, whereas other people like introverts might need to be reminded that empathy is a natural virtue of our personalities. So what are the possible ways to effectively awaken empathy in our minds, especially in the case for introverts?

First of all, we have to actively appreciate the value and benefit of showing empathy, which were discussed above. Let us not forget that as doctors, our job is to treat human beings not the mere diseases. Patients often visit doctors when they are suffering, sometimes even faced with life changing or threatening diseases. And at their most vulnerable state, we are often the only one who can provide hope. Hence, being able to be trusted by patients that we are able to handle their lives is crucial. And understanding the patients’ feelings by showing empathy is an essential first step in building trust and a therapeutic relationship.

Secondly, we need to recognise the problems that lead to the inability of showing empathy or poor performance in OSCEs. Amongst different factors, stress and nervousness are two major ones. ( Muldoon, Biesty and Smith *et al*., 2014) This is especially true with introverts, where we feel particularly uncomfortable when performing in front of people we are not acquainted with. Therefore, finding ways to control stress or going into an OSCE with minimal stress is a crucial way to maximize a student’s performance. A particularly useful way to reduce stress and improve empathy skills is by repeatedly practicing simulated doctor patient consultation scenarios, through role plays. This is a powerful method as it allows students to get accustomed to dealing with different situations in unfamiliar environments. Through progressive exposure, we are less likely to develop stress or nervousness when faced with the similar situations again. In Cognitive Behavioral Therapy, this is known as systematic desensitization. ( Sajadi *et al*., 2017) ( Rajiah and Saravanan, 2014)

The scenarios to be practiced should ideally start from simple consultations, mimicking those that doctors encounter routinely. This is because the techniques for dealing with these situations tend to be easier to grasp. It is also important to emphasise that practicing should be done in a confined and trusted environment. Being placed in a familiar environment at the initial stage allows the student to be his/ her true self when he/ she is interacting with the simulated patients. After each practice session, careful analysis and accurate feedback about the student’s performance should be made by the tutor. This allows the tutor to address any specific areas that the student is underperforming. For example, this can be about the overall rapport, professionalism, specific body gestures, facial expressions or phrases used. Initially we might have to constantly and consciously remind ourselves to express empathy. But soon by practice, empathy will be incorporated into our daily style of interaction and it will flow out naturally without even noticing it. Ultimately, the goal is to find the empathetic voice of an introvert, that is both real and sincere. Through my own experience during remediation, I discovered that I am not always certain about the simulated patient’s feelings which makes it difficult for me to empathise with them. Instead of straight away replying with a ‘borrowed’ empathetic phrases each time, I learnt that directly asking the SPs, “how are you feeling now?” or “what impact does this have on you and your life?” is a great way to explore their emotional state. In most cases, this allows the patient to genuinely express the things that concern him/her most.

Looking into the literature, it is well documented that female medical students are better in showing empathy. ( Bratek *et al*., 2015) There is also a study done that has shown negative correlation between the personality of introverts and their performance at the OSCEs. ( Shin, Kim and Lee, 2011) Being a male and an introvert certainly fitted the results of the studies above. However, there is limited evidence in the literature to fully support the connection between introversion and poor OSCE performance. Therefore, we suggest performing a research study in our medical school to determine whether there is a link between OSCE results and different personality traits including introversion. If a correlation is indeed found, it may suggest that the medical school could provide additional support in communication skills for introverted students.

## Take Home Messages

• Clinical communication is an essential part of medical education that medical students cannot belittle nor fake.
• Clinical communication skills and empathy of introverted students can be improved by role plays, systematic desensitisation and remediation.
• OSCE performance is likely to be linked with certain personality traits, more studies need to be done to confirm this proposition.

## Notes On Contributors

Joshua Lui, BSc, is a second year medical student at the St George’s, University of London Medical Programme delivered in Cyprus by the University of Nicosia.

Alexia Papageorgiou, is the Chair of the Centre of Medical Education at the University of Nicosia Medical School, Cyprus. ORCID number: 0000-0002-5346-1527
