# Supplementary material for: Empathy, Introversion and the OSCE. Reflections of a 2nd Year Medical Student
Source: MedEdPublish (2016). 2018 Nov 13;7:254. [Version 1] doi: 10.15694/mep.2018.0000254.1 (PMC10711963; doi:10.15694/mep.2018.0000254.1)
Supplement: Peer review history

# Reviewer report: Reviewer response for version 1

**DOI:** 10.21956/mep.19716.r29510

**Published:** 2019-04-30

**Authors:** Saee Deshpande

**Affiliations:** 1 Maharashtra University of Health Sciences, Nashik- VSPM Dental College Nagpur

This review has been migrated. The reviewer awarded 3 stars out of 5 very well written paper, lot of genuine feelings and efforts put in at doing background research are appreciated. one query, has the author tested his personality on any scale such as MBTI..what is the basis of labelling himself as introvert? May be personality has no role and he just needs to improve communications skills.As rightly pointed out by one of the reviewer we need to feel empathy and then show it, on simulated patients its difficult to feel..however, such training is required and it will definitely make u a better healthcare professional ultimately...my compliments and best wishes for the author

Reviewer Expertise:

NA

No decision status is available

# Reviewer report: Reviewer response for version 1

**DOI:** 10.21956/mep.19716.r29508

**Published:** 2018-11-20

**Authors:** BALAJI ARUMUGAM

**Affiliations:** 1 TAGORE MEDICAL COLLEGE AND HOSPITAL

This review has been migrated. The reviewer awarded 5 stars out of 5 Is personality one of the factors that affect the way a medical student or doctor communicates with his/ her patients?I congratulate the author for writing this article on introversion and OSCE performance. This is typical reflective writing by a second year medical student on his experience of failure in OSCE, for not showing empathy towards the simulated patients in an unfamiliar situation and being an introvert. The situation was clearly written and the factors for the failure in OSCE were expressed in depth in the article. I loved reading the reflection of a second year medical student regarding the factors like being an introvert, preoccupied mind set, simulated patients as actors and actresses. In order to fulfill his examination criteria, using the phrase and regurgitating to express empathy was practiced by the student. A person’s expression of empathy can be influenced by various factors, such as stress, his past experience, confidence or personality – well said by the author in the article. Solutions 1. A particularly useful way to reduce stress and improve empathy skills is by repeatedly practicing simulated doctor patient consultation scenarios, through role plays. 2. This is a powerful method as it allows students to get accustomed to dealing with different situations in unfamiliar environments. 3. The scenarios to be practiced should ideally start from simple consultations, mimicking those that doctors encounter routinely. over all I enjoyed reading the article and its a great experience to learn from reflection of students.

Reviewer Expertise:

NA

No decision status is available

# Reviewer report: Reviewer response for version 1

**DOI:** 10.21956/mep.19716.r29509

**Published:** 2018-11-15

**Authors:** Thomas Puthiaparampil

**Affiliations:** 1 UNIVERSITI MALAYSIA SARAWAK

This review has been migrated. The reviewer awarded 4 stars out of 5 I was touched by this article written with such sincerity and truthfulness. I consider it quite unfortunate that a high scoring medical student should fail miserably at OSCE and feel so intensely bad about it as to write an article like this. First of all, I am not convinced that such an excruciating assessment of twelve 10 minute OSCE stations should be examined for a second year medical student, who might not have completed their basic sciences and have enough clinical experience. I am also not convinced about the new trend in using actors to simulate real patients. It looks very artificial and indeed a dramatic situation, especially as it is known to the candidates. They already know what matters more is their acting talent than clinical knowledge.It is very unfortunate that an excellent student should feel disheartened because he could not perform as he lacked dramatic talents. The author has mentioned that he refused to act because he knew it is not a real situation. It will be a wrong direction of medical education to focus more on artificial empathy rather than real knowledge and human emotions. An extrovert doctor who gives more importance to impressing the patients by his acting talents rather than giving a healing touch will not be genuinely liked by his patients. An introvert is more likely to feel genuinely about his patients and be caring and eventually being appreciated by his patients.Introverts are not inferior to extroverts. There are many geniuses who were introverts. It is a fact that extroverts can do much better in acting. But sadly medical profession should not highlight acting talents. On the contrary give more credit to genuine feelings and empathy. Real concern and empathy will come only in real situations.

Reviewer Expertise:

NA

No decision status is available

# Reviewer report: Reviewer response for version 1

**DOI:** 10.21956/mep.19716.r29511

**Published:** 2018-11-14

**Authors:** Ken Masters

**Affiliations:** 1 Sultan Qaboos University

This review has been migrated. The reviewer awarded 4 stars out of 5 A well-written piece that identifies important things to consider around OSCEs.On the simulated patient’s being an actor – yes, you are quite right. This is a hurdle that needs to be overcome. Simulation is a crucial part of skills' learning in most professions. Frankly, it is the only way to determine that you could perform the required work under real-life conditions. One cannot risk finding that out for the first time in the presence of real patients.The major issue that you have highlighted, and return to several times, however, is your introversion, best expressed in these lines: “Firstly, as an introvert, I mostly enjoy spending time alone rather than interacting with others. Therefore, I always find it hard to appreciate the feelings and emotions of other people, especially when they are people that I am not acquainted with.” Because I don’t know you, it is dangerous to speculate too deeply, but your words appear to show the real problem – your feelings. The problem is not in the communication, but the struggle to connect. Again, a sentence like “And understanding the patients’ feelings by showing empathy is an essential first step in building trust and a therapeutic relationship” misses the issue. The first step is NOT to show empathy. The first step is to FEEL empathy. To show it is the NEXT step, and is an expression of what is felt. One does not understand “the patient’s feelings by showing empathy”. The two are completely disconnected. I can see why you would like to have the repeated role-plays, with guidance by the tutor, but you are narrowing the scenario too much. One cannot have 100 role-plays, and, by your suggestion, it looks like it would require that many. The problem is NOT the connection between doctor and patient. The problem is connection between person and person..Therefore, the solution that you propose (“the medical school could provide additional support in communication skills for introverted students”) would, not solve much (Yes, extra support might help, but the paper is suggesting that this kind of communication skills-support can overcome the initial problem of connection at a human level – it simply won’t). The problem is not in the communication, but in the feelings that precede it. At best, your proposal would teach students how best to fake it. At worst, it would lead to frustration and eventual dissatisfaction on your part. As a doctor working directly with patients, one’s real feelings of empathy (not merely expressing it, but feeling it) are crucial. Again, with some trepidation, I would suggest that any medical student who cannot “appreciate the feelings and emotions of other people” should visit a counsellor. Failing that, they may have choose a career that does not require this skill.So, although I pretty strongly disagree with your proposal, I also feel that this piece is well worth reading by medical educators and future medical doctors, and would probably be a value discussion text in medical education.

Reviewer Expertise:

NA

No decision status is available
